# Supplementary figures and images for: Genome-Wide Identification and Expression Analysis of the SQUAMOSA Promoter-Binding Protein-like (SPL) Transcription Factor Family in Catalpa bungei
Source: Int J Mol Sci. 2023 Dec 20;25(1):97. doi: 10.3390/ijms25010097 (PMC10779025; doi:10.3390/ijms25010097)

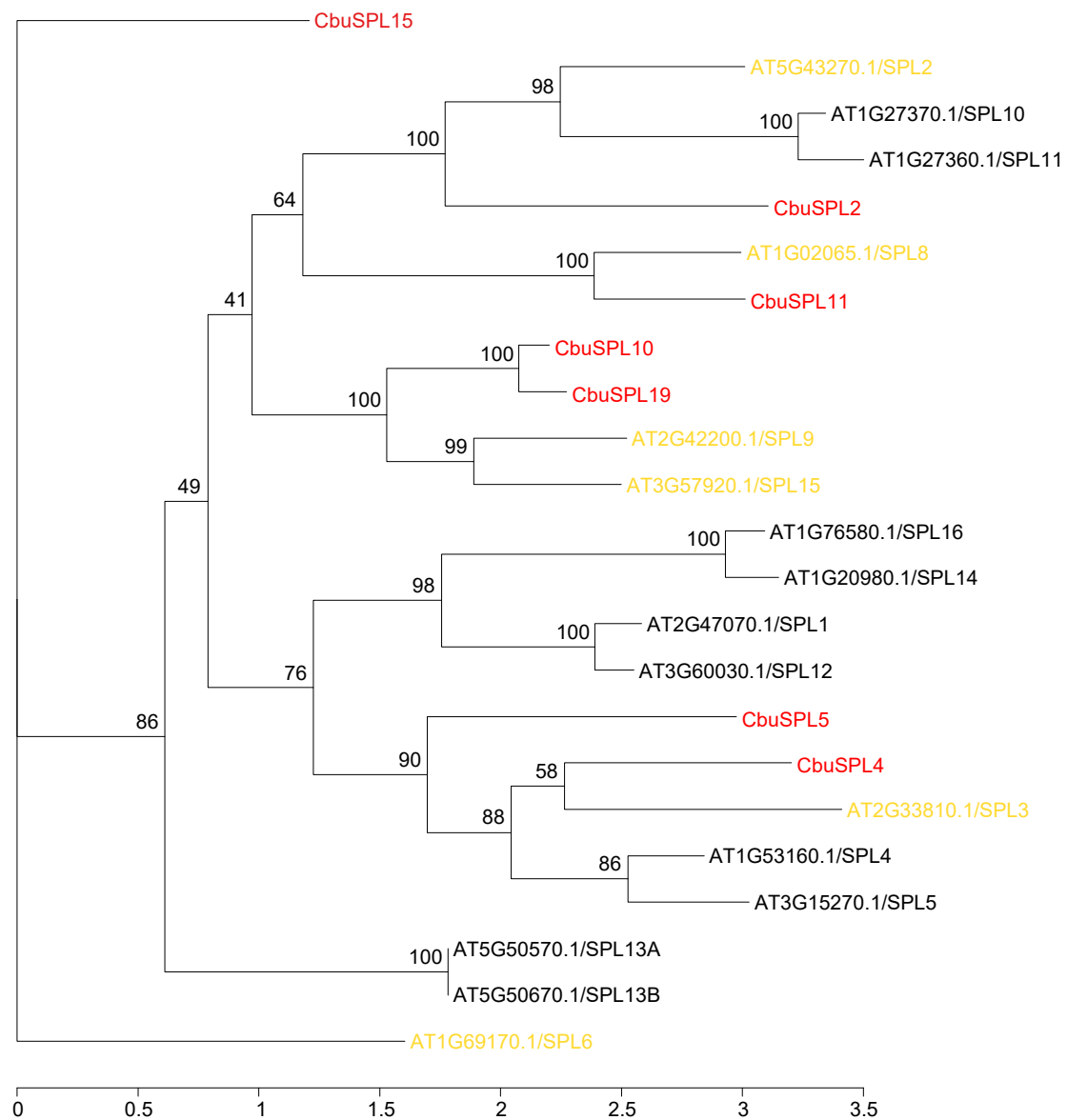

Supplementary Figure S1. The best homologous hits of *CbuSPL* genes in *Arabidopsis thaliana*.

Supplement: Supplementary file 1 [file ijms-25-00097-s001.zip › Supplementary Figure S1.pdf]
